# Antimicrobial and Antioxidant Activities of Bauhinia racemosa Lam. and Chemical Content 

**Published:** 2014

**Authors:** Khaled Rashed, Monica Butnariu

**Affiliations:** a *Pharmacognosy Department, National Research Centre, Dokki, Giza, Egypt. *; b *Chemistry and Vegetal Biochemistry, Banat's University of Agricultural Sciences and Veterinary Medicine from Timisoara, Calea Aradului, Timisoara 300645, Romania. *

**Keywords:** *Bauhinia racemosa*, Aerial parts, Antimicrobial activity, Antioxidant activity, Chemical content

## Abstract

Methanol 70% extract of *Bauhinia racemosa *aerial parts was tested for antimicrobial activity against different bacterial and fungal strains and for antioxidant activity and also total content of polyphenols with phytochemical analysis of the extract was determined. The results have showed that the extract has a significant antimicrobial activity, it inhibited the growth of *Bacillus subtilis *and also it was highly active against *Candida albicans *suggesting that it can be used in the treatment of fungal infections. The extract has a good antioxidant activity, it has shown high values of the trolox equivalent antioxidant capacity and polyphenol content while it has shown a lower value of oxygen radical absorbance capacity. Phytochemical analysis has shown that it has interesting phytochemical bioconstituents, include flavonoids, tannins and others, and the deep phytochemical study results in the isolation of methyl gallate, gallic, kaempferol, quercetin, querection 3–O–α–rhamnoside, kaempferol 3–O–β–glucoside, myricetin–3–O–β– glucoside, querection–3–O–rutinoside (Rutin). All compounds were identified by different spectroscopic analyses (UV, ^1^H–NMR, ^13^C–NMR) and Mass Spectrometry (MS).

## Introduction

Infectious diseases are the world’s leading cause of premature deaths, killing almost 50 000 people every day. Infections due to a variety of bacterial etiologic agents, such as *Staphylococcus aureus, Escherichia coli, Pseudomonas aeruginosa *and *Klebsiella pneumoniae *are most common. With the continuous use of antibiotics, microorganisms have become resistant, in addition to these problems; antibiotics are sometimes associated with adverse effects on host which include hypersensitivity, depletion of beneficial gut, mucosal microorganism, immunosuppression and allergic reactions (1, 2) has created immense clinical problem in the treatment of infection diseases (3, 4). Therefore, there is a need to search for new potential effective biocompounds against pathogenic bacteria and fungi. There have been many stretched based studies screening programmes initiated over the precedent years, in which extensive numbers of plant species have been estimated for their antimicrobial potential (5-7)*. *Free radicals concur from over a hundred from over a hundred disorders in humans through atherosclerosis, arthritis and ischemia, damage of many tissues, central nervous system (CNS) damage, gastritis, cancer and AIDS. Antioxidants or molecules with radical scavenging capacity are intent to execute a potential effect against free radical damage. It has been mentioned the antioxidant activity of plants might be due to their phenolic or/and flavonoid biocompounds (8, 9)*. Bauhinia racemosa *belongs to Caesalpiniaceae Family. It occurs in India, Ceylon and China. Many uses of the plant organs in folk medicinal were reported, flowers is used as a diuretic. Flowers, buds and dried leaves are used to treat dysentery. Root bark is used in inflammation of liver (10, 11). Seeds are tonic and aphrodisiac. Leaves have antidiabetic Action (12, 13). Plant is used in snake bite and scorpion string. Chemical bioconstituents such as β–sitosterol and β–amyrin were separated from the stem bark (14, 15), besides these biocompounds, at least two flavonols (kaempferol and quercetin) and two coumarins (scopoletin and scopolin) were separated from the leaves of the plant (16). Stilbene (resveratrol) was separated from the heart wood of *B. racemosa *(17). General phyto–pharmacological screening of the plant have revealed that the ethanol extract of *B. racemosa *leaves shows analgesic, antipyretic, anti–inflammatory and antiplasmodic activities (18, 19) as well as antimicrobial activity and antihistaminic effect (20, 13). The fresh flower buds of the plant showed antiulcer activity (21), as well as hypotensive and hypothermic activity (22, 23). No previous biological and phytochemical examinations of *Bauhinia racemosa *aerial parts have been undertaken. The purpose of the present study was to evaluate the antimicrobial and antioxidant activities of aerial parts from the plant and also total polyphenol content with phytochemical analysis were determined.

## Experimental

*Material and methods*

UV/VIS: Shimadzu UV-visible recording spectrophotometer model-UV 240 (NRC, Egypt). ^1^H-NMR spectra: Varian Unity Inova 400 (400 MHz); ^13^C-NMR spectra: Varian Unity 400 (100 MHz) (Graz University, Austria). MS (Finnigan MAT SSQ 7000, 70 ev). (Silica gel (0.063-0.200 mm for column chro matography) and Sephadex LH-20 (Pharmacia Fine Chemicals). Solvent mixtures, BAW (*n*-butanol: acetic acid: water 4:1:5 upper phase, 15% acetic acid: water: glacial acetic acid: 85:15). Paper Chromatography (PC) Whatman No.1 (Whatman Led. Maid Stone, Kent, England) sheets for qualitative detection of flavonoids and sugars.

*Plant material*

Aerial parts of *Bauhinia racemosa *were collected from Orman garden, Giza, Egypt in April 2011. The plant was identified by Dr. Mohammed El–Gebaly, Department of Botany, National Research Centre (NRC) and by Mrs. Tereez Labib Consultant of Plant Taxonomy at the Ministry of Agriculture and director of Orman botanical garden, Giza, Egypt. A voucher specimen No. 2345 is deposited in the herbarium of Orman garden, Giza, Egypt. 

*Prepartion of the extract*

700 g of air dried powder from the aerial parts of *B. racemosa *was extracted with methanol 70% at room temperature several times until exhaustion. The extract was concentrated under reduced pressure to give 54 g of crude extract**.**

*Antimicrobial assays*

The quantitative assay of the antimicrobial activity was performed by broth microdilution method (24, 25) in 96–well microplates in order to establish the minimal inhibitory concentration (MIC). The antimicrobial activity was tested against Gram–positive strains (*Staphylococcus aureus *ATCC 29213*, Bacillus subtilis *ATCC), Gram–negative (*Escherichia coli *ATCC 25922*, Pseudomonas aeruginosa *ATCC 7953*, Klebsiella pneumoniae *ATCC 10131) and fungal strain (*Candida albicans *ATCC 10231). The methanol extract of *B. racemosa *was tested for its antimicrobial activity using a qualitative screening assay of the antimicrobial properties by the adapted disk diffusion method, Kirby–Bauer method (26). The quantitative assay of the antimicrobial activity was performed by binary microdilution method (24), in order to establish the minimal inhibitory concentration (MIC). The antimicrobial activity of the investigated extract was tested against bacterial and fungal strains: Gram positive (*Staphylococcus aureus, Bacillus subtilis*), Gram–negative (*Escherichia coli, Pseudomonas aeruginosa, Klebsiella pneumoniae*) and fungal strains (*Candida albicans*). The microbial strains were identified using a VITEK I automatic system. VITEK cards for the identification and the susceptibility testing (GNS–522) were inoculated and incubated according to the manufacturer’s recommendations. In our experiments there were used bacterial suspensions of 1.5x108 UFC/ mL or 0.5 McFarland density obtained from 15–18 h bacterial cultures developed on solid media. The antimicrobial activity was tested on Mueller–Hinton medium recommended for the bacterial strains and Yeast Peptone Glucose (YPG) medium for *Candida albicans*. Solutions of the extract in DMSO (dimethyl sulfoxide) having 2048 μg/ mL concentration were used.

*Qualitative screening of the antimicrobial properties of the extract*

The antimicrobial activity of the extract was investigated by qualitative screening of the susceptibility spectrum of different microbial strains to the tested extract solubilised in DMSO (1 mg/mL) using adapted variants of the diffusion method. In the 1st variant, 10 μL of the extract solution were equally distributed on the paper filter disks placed on Petri dishes previously seeded “in layer” with the tested bacterial strain inoculums. In the 2^nd^ variant, 10 μL of the tested extract solutions were placed in the agar wells cut in the solid culture medium seeded with the microbial inoculum. In the 3^rd^ variant of the qualitative antimicrobial activity assay, 10 μL of the extract solutions were spotted on Petri dishes seeded with bacterial/yeast inoculum. In all the three variants, the Petri dishes were left at room temperature to ensure the equal diffusion of the compound in the medium or to allow the drop of solution to be adsorbed in the medium and afterwards the dishes were incubated at 37 °C for 24 hours. The solvent used was also tested in order to evaluate a potential antimicrobial activity.

*Quantitative assay of the antimicrobial activity*

For the quantitative assay of the antimicrobial activity of the extract by the microdilution method (24, 27) in liquid medium distributed in 96–well plates, binary serial dilutions of the tested extract solutions were performed. There were obtained concentrations from 1000 μg/mL to 0.97 μg/mL in a 200 μL culture medium final volume, afterwards each well was seeded with a 50 μL microbial suspension of 0.5 MacFarland density. In each test a microbial culture control (a series of wells containing exclusively culture medium with the microbial suspension) and a sterility control (a series of wells containing exclusively culture medium) were performed. The plates were incubated for 24 hours at 37 °C.

*Antioxidant assays*

Extraction: 0.2 g of extract with 10 mL Millipore water boiled was sonic, centrifuged and filtered. Evaluation of antioxidant activity of the extract, using methods Oxygen Radical Absorbance Capacity (ORAC), Trolox equivalent antioxidant capacity (TEAC) and determination of total polyphenols content:

*Oxygen Radical Absorbance Capacity (ORAC)*

This method determines peroxil radical inhibition capacity, inducing oxidation highlighting the classical radical release; H atom transfer ORAC values were reported as Trolox equivalents, is expressed as micromol TE/DW. The intensity was monitored at 485 nm and 525 nm for 35 min.

*Trolox equivalent antioxidant capacity method (TEAC)*

This method is based the neutralizing capacity the radical anion ABTS^+^ [2,2'–azino–bis (3–ethylbenzothiazoline–6–sulphonic acid)] by antioxidants. ABTS is oxidized by radicals peroxil or other oxidants to its radical cation ABTS^+^, intensely colored (λmax = 734 nm). Antioxidant capacity is expressed compounds tested as potential, to discoloration by direct reaction with it radical ABTS^+^.

*The total content of polyphenols*

The blue compounds formed between phenols and Folin–Ciocalteu reagent phenolic compounds are independent of structure, thus developing complex between metal center and phenolic compounds. Absorption was recorded at a wavelength of 765 nm. Total phenol content was expressed as gallic acid equivalents (28).

*Qualitative phytochemical analysis*

The extract was tested for the presence of bioactive compounds by using following standard tests (Molisch᾽s test for carbohydrates, Shinoda test for flavonoids, froth test for saponins, Salkowski᾽s for terpenes and sterols, FeCl_3_ and Mayer᾽s reagents for detecting of tannins and alkaloids, respectively (29-31).

*Isolation of bioactive compounds from methanol extract of B. racemosa*

The extract 54 g was defatted with *n*–hexane and the extract residue 42 g which was subjected to Silica gel column chromatography eluting with dichloromethane, ethyl acetate and methanol gradually. One hundred and twenty fractions were collected. The fractions that showed similar Paper Chromatography (PC) in Butanol–Acetic acid–Water 4:1:5 (BAW) and 15% acetic acid were combined to give 4 fractions (I, II, III, and IV). Fraction I (950 mg) was subjected to sub–column of silica gel eluted with dichloromethane: ethyl acetate (60:40) gave compound 1 (methyl gallate, 60 mg) and elution with dichloromethane: ethyl acetate (90:10) gave compound 2 (gallic acid, 100 mg). Fraction II (830 mg) was subjected to sub–column of silica gel eluted with dichloromethane: ethyl acetate (95:5) to give compound 3 (kaempferol, 40 mg) and elution with ethyl acetate solvent gave compound 4 (quercetin, 25 mg). Compound 5 (quercetin–3–O–α–rhamnoside, 35 mg) and 6 (kaempferol–3–O–β–glucoside, 15 mg) were obtained from elution with ethyl acetate: Methanol (90:10) from fraction III (640 mg) and their purification were carried out on sephadex LH–20 column which eluted with methanol (50%). Compound 7 (myricetin–3–O–β–glucoside, 20 mg) and 8 (quercetin–3–O–rutinoside, 60 mg) were isolated from fraction IV (1.2 g) by sub–column of silica gel eluted with ethyl acetate: methanol (60:40) and their purification were carried out on sephadex LH–20 column which eluted with methanol (50%).

*Acid hydrolysis of flavonoids*

Solutions of 5 mg of compounds 5, 6, 7 and 8 in 5 mL 10% HCl were heated for 5 h. The reaction mixture was extracted with Ethyl acetate. The Ethyl acetate fraction (aglycone) and the aqueous fraction (sugars) were concentrated for identification. The sugars were identified by TLC (acetonitrile–water 85:15) by comparsion with authentic samples.

## Results and Discussion

The present investigation was focused to evaluate the antimicrobial activity (expressed in μg/mL) and antioxidant capacity (expressed as Trolox equivalents and total polyphenol content) as can be seen in Table 1 and Table 2. The aim of the present study was to investigate the presence of phytochemicals*, *estimation of the main bioactive constituents of *B. racemosa *aerial parts. The major bioactive components of *B. racemosa *are methyl gallate, gallic acid, kaempferol, quercetin, quercetin 3–O–α–rhamnoside, kaempferol 3–O–β–glucoside, myricetin 3–O–β–glucoside and quercetin 3–O–rutinoside. The structure of bioactive components was elucidated by different spectroscopic analyses.

**Table 1:** Results of Antimicrobial activity of *B. racemosa *extract expressed in μg/ mL (MIC).

**Material tested**	***K. pneumoniae*** **IC 13420**	***E. coli*** **IC 13529**	***S. aureus*** **IC 13204**	***P. aeruginosa*** **ATCC 27853**	***B. subtil*** **ATCC 6633**	***C. albicans*** **IC 249**
*B. racemosa extract*	62.5	125	250	125	31	7.8
Blank DMSO	125	125	250	125	125	125

* MIC =minimal inhibitory concentration.

**Table 2:** Results of Antioxidant capacity of *B. racemosa *extract tested were expressed as Trolox equivalents and total polyphenol content

**Material tested**	**Total polyphenol content (gallic acid mg/g DW)**	**Oxygen radical absorbance capacity (ORAC) assay value**	**Trolox equivalent antioxidant capacity (TEAC) assay value**
*B. racemosa extract*	695.1 ± 3.56 mg/g	1033 mM TE/g	201 ± 3.6 mM TE/g

**Figure 1:**
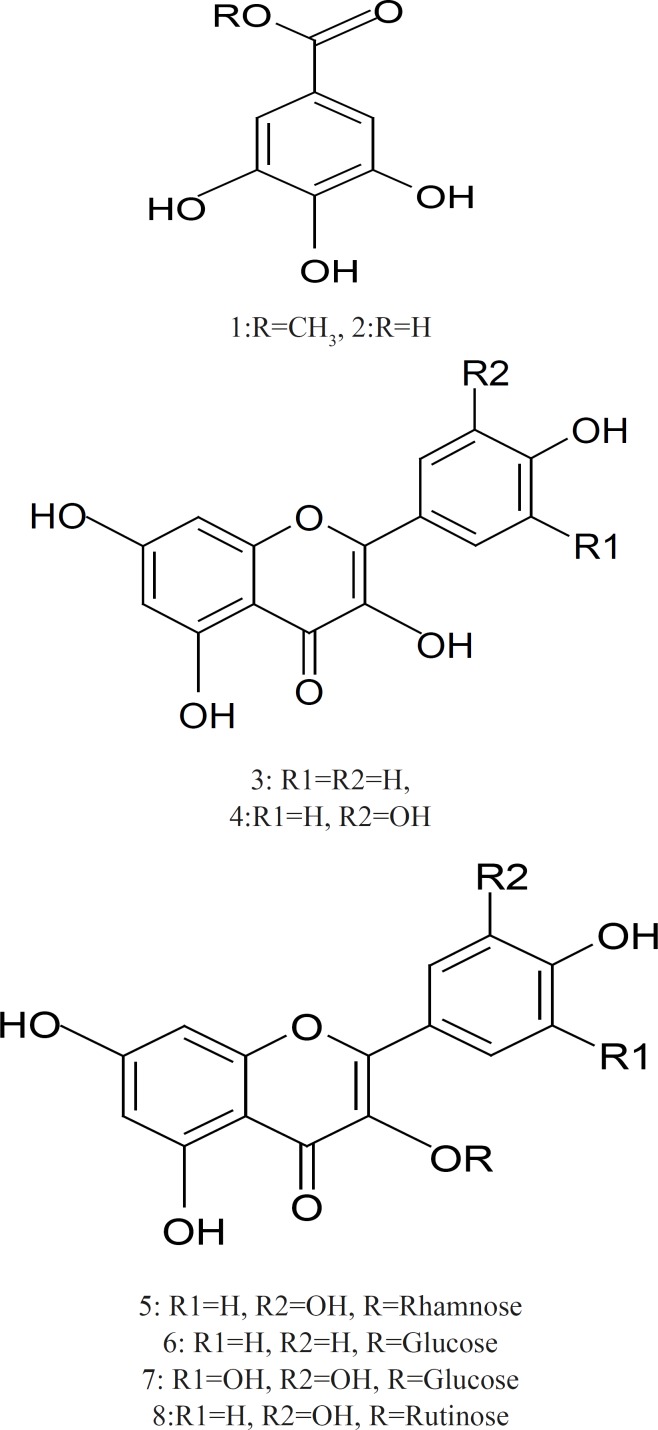
Chemical structures of Phenolic compounds isolated from *B. racemosa*

*Structure Elucidation of the isolated compounds*

*Methyl gallate *(1): white amorphous powder: UV λmax (MeOH): 275. ^1^H–NMR (DMSO–d_6_, 400 MHz): δ 6.94 (2H, s, H–2,6), 3.73 (3H, s,–OCH_3_). ^13^C–NMR (DMSO–d_6_, 100 MHz): δ 166.8 (–COO), 146 (C–3,5), 138.9 (C–4), 119.8 (C–1), 109 (C–2,6), 52 (–OCH_3_).

*Gallic acid *(2): white amorphous powder. UV λmax (MeOH): 273. ^1^H–NMR (DMSO–d_6_, 400 MHz): δ 7.15 (2H, s, H–2,6).^13^C–NMR (DMSO–d_6_, 100 MHz): δ 167.2 (–COOH), 145 (C–3,5), 137.7 (C–4), 121 (C–1), 109.1 (C–2,6).

*Kaempferol *(3): Yellow powder. UV λmax (MeOH): 265, 320, 366; (NaOMe): 276, 317, 406; (AlCl_3_): 262sh, 269, 310sh, 367; (AlCl_3_/HCl): 263sh, 268, 320sh, 344, 425; (NaOAc): 274, 306, 382; (NaOAc/H_3_BO_3_): 267, 368. 1H–NMR (DMSO–d_6_, 400 MHz): δ 8.11 (2H, d, *J *= 8 Hz, H–2',6'), 6.96 (2H, d, *J*=8 Hz, H–3',5'), 6.47 (1H, d, *J *= 2 Hz, H–8), 6.19 (1H, d, *J *= 2 Hz, H–6). EI–MS: *m/z *286.

*Quercetin *(4): Yellow powder. UV λmax (MeOH): 255, 267, 371; (NaOMe): 270, 320, *Citrus Aurantium & Labor Pain *1077 

420; (AlCl_3_): 270, 455; (AlCl_3_/HCl): 264, 303sh, 315sh, 428; (NaOAc): 257, 274, 318, 383; (NaOAc/H_3_BO_3_): 259, 387. 1H–NMR (DMSO–d6, 400 MHz): δ 7.74 (1H, d, *J *= 8, 2 Hz, H–2'), 7.55 (1H, d, *J *= 2 Hz, H–6'), 6.92 (1H, d, *J *= 8 Hz, H–5'), 6.42 (1H, d, *J *= 1.2 Hz, H–8), 6.15 (1H, d, *J *= 1.2 Hz, H–6). EI–MS: *m/z *302.

*Q*uercetin 3–O–α–rhamnoside (5) Yellow crystals: 1H–NMR (400 MHz, DMSO–d_6_) δ ppm 7.26 (2H, m, H–2'/6'), 6.83 (1H, d, J=9 Hz, H–5'), 6.49 (1H,d, *J*=2.5 Hz, H–8),6.14(1H, d, J=2.5Hz,H–6), 5.25 (1H, br s, H–1'') 0.78 (3H, d, *J*=6Hz). ^13^C–NMR (100 MHz, DMSO–d_6_): δ ppm 177.42 (C–4), 167.45 (C–7), 161.40 (C–5), 157.01 (C–2), 157 (C–9), 149.19 (C–4'), 145.57 (C–3'), 134.12 (C–3), 131.97 (C–6'), 121.40 (C–1'), 115.71 (C–2'), 115.40 (C–5'), 103.10 (C–10), 101.97 (C–1''), 99.98 (C–6), 94.47 (C–8), 71.47 (C–4''), 70.94, 70.85, 70.62 (C–2'', C–5'', C–3''), 17.78 (C6'').

*Kaempferol 3–O– β–glucoside (6): *Yellow crystals. UV λmax (MeOH): 266, 364; (NaOMe): 274, 327sh, 401; (AlCl_3_): 274, 304, 349, 396; (AlCl_3_/HCl): 274, 345, 394; (NaOAc): 274, 305, 393; (NaOAc/H_3_BO_3_): 267, 352. 1H–NMR (400 MHz, DMSO–d_6_) δ 8.0 (2H, d, H–2'/6', *J*=8.5), δ 6.9 (2H, d, H–3'/5', *J*=8.5), δ 6.5 (1H, d, *J*=2 Hz, H–8), 6.2 δ (1H,d, *J*=2.5 Hz, H–6), 5.4 (1H,d,*J*=7.5, H–1''), 3.80–3.10 (5H,m,remaining sugar protons).

*Myricetin 3–O–β–glucoside *(7): 1H–NMR of (400 MHz, DMSO–d_6_) δ 7.16 (2H, s, H–2'/6'), δ 6.13(1H, d, *J*=2.5 Hz, H–6), 6.35 δ (1H,d, *J*=2.5 Hz, H–8), 5.45 (1H, d, *J*=7.55, H–1''), 3.90–3.20 (m, remaining sugar protons). 13C–NMR (100 MHz, DMSO–d_6_): δ ppm 177.85 (C–4), 164.83 (C–7), 161.71 (C–5), 156.81 (C–2), 156.71 (C–9), 146.49 (C–3'), 145.87 (C–5'), 137.97 (C–4'), 133.95 (C–3), 120.49 (C–1'), 109 (C–2',6'), 104.37 (C–10), 101.4 (C–1''), 99.22 (C–6), 93.00 (C–8), 78.04 (C–5''), 77.04 (C–3''), 74.44 (C–2''), 70.36(C–4'') 61.52 (C–6'').

*Quercetin 3–O–rutinoside *(Rutin) (8): 1H–NMR (400 MHz, DMSO–d6): δ ppm 7.54 (2H, m H–2'/6'), 6.85 (1H, d, J=9 Hz, H–5'), 6.38 (1H, d, *J*=2.5Hz, H–8), 6.19 (1H,J=2.5Hz, H–6), 5.35 (1H, d, J=7.5 Hz, H–1''), 5.33 (1H, br s, OH), 5.02,(2H each, br s, OH groups), 4.39 (1H, s, H–1'''), 3.90–3.20 (m, remaining sugar protons), 0.99 (3H, d, J=6 Hz, H–6'''). ^13^C NMR(100 MHz, DMSO–d_6_): δ ppm 177.85 (C–4), 164.70 (C–7), 161.68 (C–5), 157.14 (C–2), 156.95 (C–9), 148.92 (C–4'), 145.25 (C–3'), 133.76 (C–3), 122.12 (C–6'), 121.66 (C–1'), 116.73 (C–2'),115.72 (C–5'),104.41 (C–10), 101.66 (C–1'''), 101.23 (C–1''), 99.24 (C–6), 94.16 (C–8), 74.58(C–3''),72.33(C–5''), 72.2 (C–4'''), 71.05(C–2''), 70.8(C–2'''), 70.87(C–3''') 70.49(C–4'') 68.74 (C–6'') 18.19(C–6''').

*Antimicrobial activity of B. racemosa extract*

For the antimicrobial qualitative methods, *i.e. *paper filter disks impregnated with the tested extract solution and disposal of the respective solutions in agar wells, the reading of the results was performed by measuring the microbial growth inhibition zones around the filter disks impregnated with the testing extract and around the wells, respectively. The most efficient qualitative method proved to be the direct spotting of the tested solutions on the seeded medium, the results being very well correlated with the results of the (minimal inhibitory concentration) MIC quantitative assay. For the quantitative methods of the antimicrobial activity of the tested extract by the microdilution method in liquid medium, the MIC was read by wells observation: in the first wells containing high concentrations of extract, the culture growth was not visible, the microbial cells being killed or inhibited by the tested extract. At lower concentrations of the tested extract, the microbial culture becomes visible. The lowest concentration which inhibited the visible microbial growth was considered the MIC (μg/mL) value for the extract. In the next wells, including the standard culture growth control wells, the medium become muddy as a result of the microbial growth. In the sterility control wells series, the medium had to remain clear. From the last well without any visible microbial growth and from the first one that presented microbial growth, Gram stained smears were performed for the results confirmation. In Table 1, there are the results of the quantitative assay of the antimicrobial activity of the *B. racemosa *extract. Our results have shown that the extract was highly active against *C. albicans, *suggesting its possible use in the treatment of fungal infections, also it exhibited antimicrobial activity on *B*. *subtilis *and it has shown a moderate antimicrobial activity against *K*. *pneumoniae *but it was not active on other bacterial strains*. *

*Antioxidant activity B. racemosa and Total polyphenol content*

Antioxidant activity was evaluated by Oxygen Radical Absorbance Capacity (ORAC) and Trolox equivalent antioxidant capacity method (TEAC) assays. In (TEAC) assay, *B. racemosa extract *showed high TEAC value 201 ± 3.6 mM TE/g (Table 2), as well as it showed a high total polyphenol content 695.1 ± 3.56 mg/g (Table 2) which was expressed as gallic acid equivalents, while in ORAC assay, *B. racemosa *extract has shown lower ORAC value (1033 mM TE/g) (Table 2) these results suggest the antioxidant activity of *B. racemosa *and the high content of polyphenols in the methanol extract of *B. racemosa *is in agreement with phytochemical analysis of the extract which has shown the presence of flavonoids, tannins, coumarins (phenolic components), alkaloids and carbohydrates (Table 3). Phenolic compounds form one of the main classes of secondary metabolites. They display a large range of chemical structures and are responsible for the major bioactivity of plants. Flavonoids have shown a significant antimicrobial activity (32, 33), tannins have shown a significant antimicrobial and antioxidant activities (34), as well as coumarins have shown a good antimicrobial and antioxidant effects (35). 

Chemical and Chromatographic separations of *B. racemosa *methanol extract yielded eight known phenolic compounds which were isolated and purified by standard methods. Compounds 1, 2 were white amorphous powder, showed chromatographic properties and colour reactions (positive FeCl_3_ and KIO_3_ tests) indicative of galloyl esters (36). Compounds 3, 4 detected as yellow spots on PC under UV light did not change by ammonia vapour*. *While compounds 5, 6, 7 and 8 appeared as dark purple spots under UV light, change to yellow when fumed to ammonia. The chemical investigation of compounds 5, 6, 7 and 8 was followed by paper chromatography to identify the hydrolytic flavonoid–*O*–glycoside products whether aglycone and sugar moieties. The identification of the isolated compounds was confirmed by co–chromatography with authentic samples, UV and NMR spectroscopy and MS spectrometry. The spectral data of the isolated compounds were compared with the literature data (37-39). On the basis from above, it can be concluded that *B. racemosa *methanol extract has significant antimicrobial activity and possess better antioxidant activity and these activities are due to the high polyphenol content and the interesting bioactive compounds include flavonoids (kaempferol, quercetin, querection–3–O–α–rhamnoside, kaempferol–3–O–β–glucoside, myricetin–3–O–β–glucoside and querection–3–O–rutinoside) and tannins (methyl gallate and gallic acid).
